# Surgical management of consecutive multisegment thoracic and lumbar tuberculosis: anterior-only approach vs. posterior-only approach

**DOI:** 10.1186/s13018-020-01876-3

**Published:** 2020-08-20

**Authors:** Chen Zhao, Lei Luo, Liehua Liu, Pei Li, Lichuan Liang, Yongjian Gao, Fei Luo, Jianzhong Xu, Qiang Zhou

**Affiliations:** 1 Department of Orthopedics, The Third Affiliated Hospital of Chongqing Medical University, Chongqing, China; 2 Department of Orthopedics, Southwest Hospital, The Army (Third Military) Medical University, Chongqing, China

**Keywords:** Spinal tuberculosis, Consecutive multisegment, Kyphosis, Posterior-only approach, Anterior-only approach, Deformity correction

## Abstract

**Purpose:**

To compare the efficacy, safety, and technical characteristics of anterior-only and posterior-only approach surgeries for the treatment of consecutive multisegment thoracic and lumbar tuberculosis.

**Methods:**

Thirty-five patients who developed consecutive multisegment thoracic and lumbar tuberculosis from September 2012 to May 2016 were retrospectively analyzed. Group A was the posterior-only surgery group, and group B was the anterior-only surgery group. The data on the surgery, deformity correction, functional scores, and complications were compared between the two groups.

**Results:**

There was no significant difference in the operation time or blood loss between groups A and B (*P* > 0.05). The preoperative average Cobb angle of kyphosis in groups A and B were 36.2 ± 15.2° and 27.9 ± 7.7°, respectively, which significantly decreased to 4.9 ± 11.8° and 10.4 ± 5.6° after the operation, respectively (*P* < 0.05). At the final follow-up, the angles were 7.1 ± 10.5° and 14.6 ± 8.0°. The correction angle and correction rate in group A (31.3 ± 16.6°, 88.6 ± 43.6%) were greater than those in group B (17.5 ± 4.4°, 64.9 ± 14.0%) (*P* < 0.05). There was no significant difference in the loss angle between groups A and B (*P* > 0.05), but the loss rate in group B (24.0 ± 27.8%) was higher than that in group A (9.6 ± 10.2%) (*P* < 0.05). There was no significant difference in the incidence of complications between the two groups (*P* > 0.05).

**Conclusion:**

The posterior-only and anterior-only approaches can lead to satisfactory clinical results in the treatment of patients with consecutive multisegment thoracic and lumbar tuberculosis. With posterior-only surgery, kyphosis can be better corrected, and the correction can be better maintained than with anterior-only surgery.

## Introduction

After 20 years of tuberculosis (TB) prevention and treatment efforts, the incidence of TB has decreased significantly; however, the incidence is still high in economically underdeveloped areas in China [1]. At present, China is the country with the second largest number of new TB cases worldwide [2]. Spinal TB is the most common extrapulmonary TB and most cases involve a single segment; more than one segment is rarely involved [3].

The incidence of TB is highest in the thoracic spine, followed by the lumbar spine. Thoracic and lumbar TB mostly damages the anterior column of the spine, and the collapse of the anterior column leads to kyphosis [4]. Multisegment vertebral destruction increases the risk of kyphosis and neurologic impairment [5, 6]. Therefore, more multisegment cases than single-segment spinal TB cases require surgical treatment.

Multisegment thoracic and lumbar cases are difficult to treat because of the extensive lesions, neurologic impairment, and severe kyphosis. Anterior surgery is not suitable for long segmental fixation and the correction of severe kyphosis. Therefore, combined anterior and posterior surgery is recommended for cases that need long segmental fixation and the correction of severe kyphosis [7]. However, combined anterior and posterior surgery increases the surgical trauma and risk of complications [8].

In recent years, the posterior-only approach has led to satisfactory clinical results for the treatment of thoracic and lumbar TB and has been gradually accepted by surgeons [9, 10]. However, there are few reports about the application of the posterior-only approach in the treatment of multisegment thoracic and lumbar TB. The main controversy is the safety of its application. Therefore, this study retrospectively analyzed 35 cases of multisegment thoracic and lumbar TB to evaluate the safety, effectiveness, and technical features of the anterior-only and posterior-only surgical methods.

## Materials and methods

### The general data

The inclusion criteria were as follows: (1) thoracic and lumbar spinal activity TB; (2) a lesion involving more than 2 segments (more than 3 vertebrae); (3) history of anterior-only or posterior-only surgical treatment in our spinal center; and (4) an age older than 18 years.

The exclusion criteria were as follows: (1) inconsecutive multisegment thoracic and lumbar TB; (2) recurrence spinal TB; (3) TB combined with severe osteoporosis; and (4) TB combined with diseases that affect clinical observations, such as lumbar disc herniation.

This study is a retrospective study and has been approved by the Hospital Ethics Committee. We retrospectively analyzed spinal TB patients treated in our hospital from September 2012 to May 2016. A total of 35 patients were included in this study for statistical analysis. They were divided into two groups based on surgical treatment. Nineteen patients underwent posterior-only approach surgery (Group A) and sixteen patients underwent anterior-only approach surgery (Group B). General clinical information of the patients is shown in Table 1. There was no statistical difference in general information(*P* > 0.05) except age.

**Table 1:** General data

	Group A	Group B	*P* values
Patients (No.)	19	16	
Sex (male/female)^a^	8/11	8/8	*P* > 0.05
Age (y/o)	35.0 ± 11.8	50.2 ± 14.4	*P* < 0.05
Average number of segments (NO.)^a^	2.7 ± 0.8	2.3 ± 0.6	*P* > 0.05
Cases of psoas or iliac abscess (No.)^a^	5	1	*P* > 0.05
Cases of kyphosis (No.)^a^	16	14	*P* > 0.05
Cases of neurological impairment (No.)^a^	13	12	*P* > 0.05

^a^There were no significant differences in the average number of lesion-affected segments, sex distribution or number of cases with neurological impairment, kyphosis, or psoas or iliac abscess between the two groups (*P* > 0.05)

### Preoperative preparation

Upon admission, the patients underwent a routine examination related to anesthesia-related examinations. X-ray, 3-D reconstruction computed tomography (CT), and magnetic resonance imaging (MRI) examinations were performed in all patients to identify the regions of spinal TB, kyphosis, pedicle integrity, and spinal stability. The patients received anti-TB treatment for at least 2 weeks before surgery. The anti-TB treatment doses consisted of 0.3 g oral quaque die (QD) of isoniazid, 0.45 g oral QD of rifampicin, 0.75 g oral QD of ethambutol, and 0.5 g ter in die (TID) of pyrazinamide, and Levofloxacin 0.2 g intravenously (IV) bis in die (BID) was given during hospitalization. Surgical treatment was considered when the symptoms and nutritional status of the patients were improved and the erythrocyte sedimentation rate (ESR) and C-reactive protein (CRP) were decreased. During preoperative preparation, if the patient’s neurological impairment was aggravated, surgical treatment was performed early.

### Surgical technique

#### Posterior-only approach

After general anesthesia was induced, the patient was placed in a prone position. The posterior median approach was used to expose the bilateral lamina and facet joints. A posterior pedicle screw system was used for internal fixation, and the fixation area was 1–2 adjacent normal vertebral bodies if necessary. According to the lesion region, unilateral or bilateral facet joints and transverse costal processes were excised, and the intervertebral, paraspinal, and intraspinal TB lesions were completely removed with the transforaminal and/or paraspinal approach. The autologous bone, interbody fusion cage, or titanium mesh were used for intervertebral structural support according to the extent of the bone defect. The bone graft materials were mixed with isoniazid and rifampicin. Kyphosis was further corrected by additionally performing 1–2 grade osteotomy in patients with kyphosis of the spine that remained more than 10° after intervertebral structural bone grafting. The autogenous and allograft bone grafts were used to reconstruct the lamina and perform bone grafting. The psoas or iliac abscess was cleared by the anterior extraperitoneal approach during the same surgery.

#### Anterior-only approach

After general anesthesia, the patient was placed in the lateral position so that the side with the severe lesion was facing upward. A posterolateral incision was made to expose the lateral side of the affected vertebra. The ribs and transverse process of the diseased vertebra were resected, and the intervertebral, paravertebral, and intraspinal lesions were completely removed from the side of the vertebral body. Internal fixation was performed with screw-rod system, and the fixation area was 1–2 adjacent normal vertebral bodies if necessary. The autologous bone, interbody fusion cage, or titanium mesh were used for intervertebral structural support according to the extent of the bone defect. Moreover, vertebral lateral bone grafting was performed. The bone graft materials were also mixed with isoniazid and rifampicin.

### Postoperative management

The patients received treatment to prevent infection after the operation. The anemia and hypoproteinemia were treated if necessary. The drainage tube was removed after more than 7 days, and the drainage rate was less than 10 ml/24 h. One week after the operation, patients could wear a brace and ambulate gradually. Patients were required to wear the brace for 3–6 months. All patients continued to receive anti-TB therapy for 12 to 18 months after surgery, with the same doses as before surgery. If patients’ ESR and CRP did not decrease to within normal limits within 3 months, levofloxacin was additionally administered orally until the levels decreased to within normal limits. The criteria for anti-TB therapy were a therapy duration of more than 1 year, normal ESR and CRP levels, bone fusion evident in the CT scan, and non-TB lesions shown in the MRI scan.

### Statistical analysis

Patients’ clinical and imaging data were recorded. The kyphosis angle was measured by the Cobb angle. Neurological function was assessed using the American spinal injury association (ASIA) grade.

IBM SPSS 19.0 was used for statistical analysis of the data in this study. The operation time, blood loss, and kyphosis correction and loss were compared between groups, and the visual analogue scale (VAS) and Oswestry disability index (ODI) scores were compared between the preoperative and final follow-up times by the *T* test. The kyphosis angle of pre-operation, post-operation, and final follow-up times was compared by ANOVA in each group. The non-normally distributed data were transformed before analysis. The incidence of complications and improvement in neurological function were compared by the chi-square test.

## Results

All patients underwent surgery successfully without serious complications. In group A, 5 of the 19 patients had the abscess removed by a small anterior incision. The average operative time of group A was 338.4 ± 73.7 min, while that of group B was 301.1 ± 30.1 min. The average blood loss of group A was 1352.6 ± 593.8 ml, while that of group B was 1037.5 ± 377.5 ml. There was no significant difference in the operation time or blood loss between groups A and B (*P* > 0.05).

The patients were followed up for at least 2 years, with an average follow-up time of 37.9 ± 11.4 m (24–61 m). At the last follow-up, all of the patients were cured, with no complications related to internal fixation or recurrence of TB. The mean preoperative VAS scores in group A and group B were 3.7 ± 1.1 and 3.8 ± 0.8, respectively. After the operation, the mean scores decreased significantly to 1.2 ± 0.9 and 1.2 ± 0.5 (*P* < 0.05). At the last follow-up, they were 0.8 ± 0.7 and 1.1 ± 0.9. The mean preoperative ODI were 24.7 ± 12.7% and 31.2 ± 13.7% in groups A and B, respectively. At the last follow-up, they improved significantly to 7.3 ± 7.8% and 11.3 ± 6.9% (*P* < 0.05).

Before surgery, thirteen patients in group A were considered to have ASIA grade D neurological impairment, while in group B, 1 had grade B impairment, and 11 had grade D impairment. The neurological impairment cases in group A and group B significantly improved postoperatively (*P* < 0.05). In group B, 1 patient with grade B impairment recovered to grade D after surgery, while the other patients with neurological impairment in both groups improved to grade E. There was no significant difference between the two groups (*P* > 0.05).

There were 16 and 14 cases with kyphosis in groups A and B, respectively. The kyphosis corrections of group A and group B are shown in Table 2. There was no statistically significant difference in the kyphosis angle between groups A and B before the operation (*P* > 0.05). The kyphosis angles of the two groups were significantly improved after surgery (*P* < 0.05). There was no significant difference between after surgery and the last follow-up in group A (*P* > 0.05), while there was a significant difference in group B (*P* < 0.05).

**Table 2:** Kyphosis correction data

Group	No.	FU (M) ^a^	Kyphosis angle (°)			CA (°) ^c^	CR (%)^c^	LA (°)^d^	LR (%)^c^
			Pre ^a^	Post ^b^	FFU				
A	16	41.3 ± 13.1	36.2 ± 15.2	4.9 ± 11.8	7.1 ± 10.5	31.3 ± 16.6	88.6 ± 43.6	2.1 ± 2.9	9.6 ± 10.2
B	14	33.9 ± 7.7	27.9 ± 7.7	10.4 ± 5.6	14.6 ± 8.0	17.5 ± 4.4	64.9 ± 14.0	3.0 ± 1.6	24.0 ± 27.8

*FU* follow-up, *M* month, *Pre* preoperative, *Post* postoperative, *FFU* final follow-up, *CA* correction angle, *CR* correction rate, *LA* loss angle, *LR* loss rate

^a^There was no statistically significant difference in the preoperative kyphosis angle or follow-up time between the two groups (*P* > 0.05)

^b^The kyphosis angles of the two groups were significantly improved after surgery (*P* < 0.05)

^c^The kyphosis correction angle and rate in group A were greater than those in group B (*P* < 0.05). The loss rate in group B was greater than that in group A (*P* < 0.05)

^d^There was no significant difference in the loss angle between group A and group B (*P* > 0.05)

The average kyphosis correction angle and rate were 31.3 ± 16.6° and 88.6 ± 43.6% in group A and 17.5 ± 4.4° and 64.9 ± 14.0% in group B, respectively. The kyphosis correction angle and rate in group A were greater than those in group B (*P* < 0.05). The mean kyphosis correction loss angle and rate were 2.1 ± 2.9° and 9.6 ± 10.2% in group A and 3.0 ± 1.6° and 24.0 ± 27.8% in group B, respectively. There was no significant difference in the mean kyphosis correction loss angle between group A and group B (*P* > 0.05), while there was a significant difference in the correction loss rate (*P* < 0.05).

Five patients in group A developed complications, with an incidence of 26.3%, and 6 patients in group B developed complications, with an incidence of 37.5%. There was no significant difference in the incidence of complications between the two groups (*P* > 0.05). In group A, four patients with cerebrospinal fluid leakage were cured after symptomatic treatment, and one patient with incision infection was cured after anti-infection treatment. In group B, 3 patients with pleural effusion, 1 patient with pneumonia, and another patient with intercostal neuralgia were cured after conservative treatment. One patient’s lower extremity muscle strength decreased to grade 2–3 after the operation, an exploratory operation was performed immediately, and there were no noticeable abnormalities in the operative region. After the operation, the patient was treated with anti-inflammatory, dehydration, nutritive nerve, and functional exercise treatments, and the muscle strength of the lower limbs returned to normal after 6 months.

**Fig. 1:**
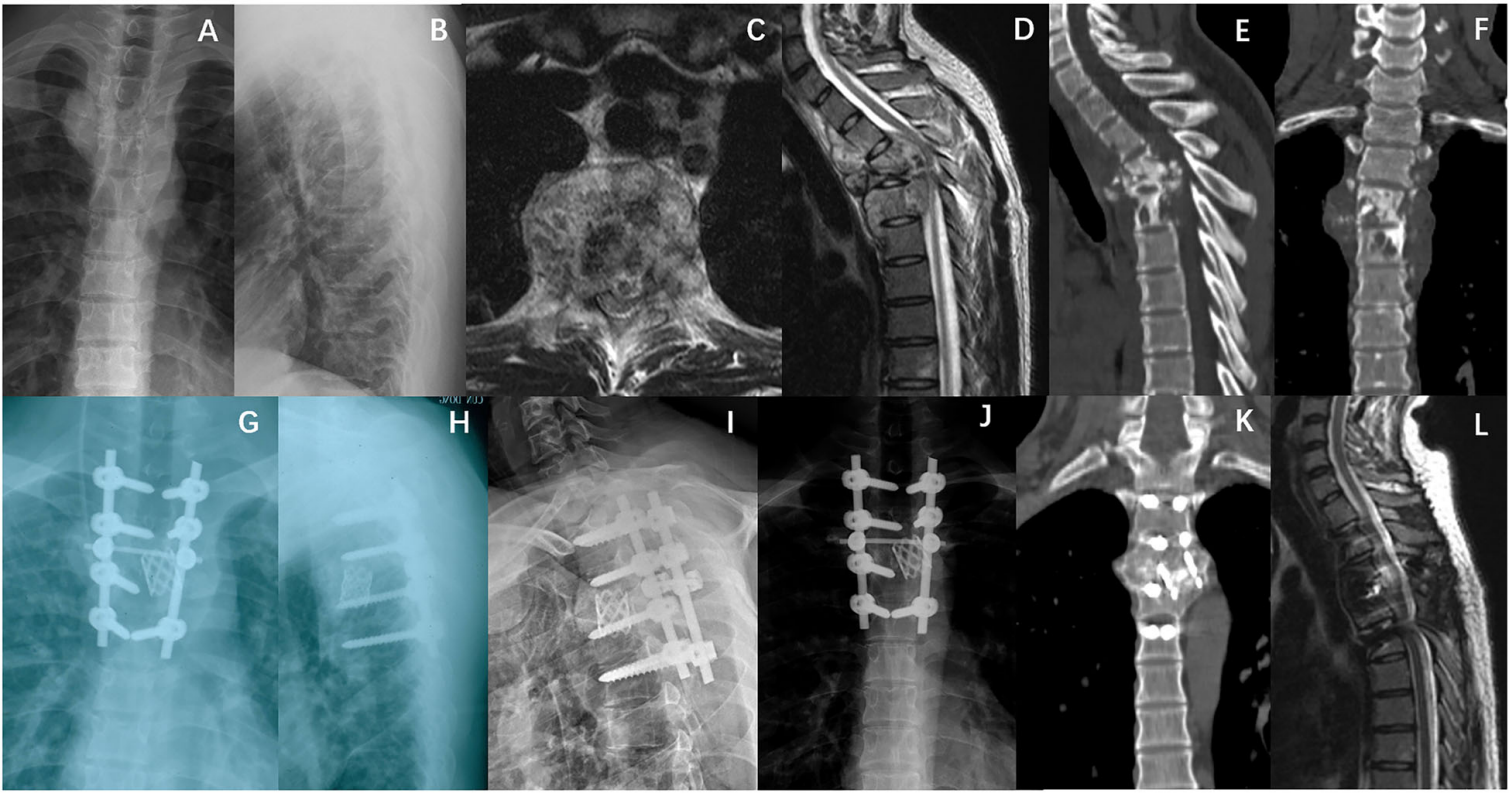
Case I. A 25-year-old male patient with T3–5 TB who was assigned to group A. **a–f** show the preoperative X-ray, CT, and MRI scans. The patient was treated with posterior-only surgery, and the internal fixation region was T3–6. **g** and **h** show the 1-week postoperative X-rays. **i–l** show the 2-year postoperative X-ray, CT, and MRI scans

**Fig. 2:**
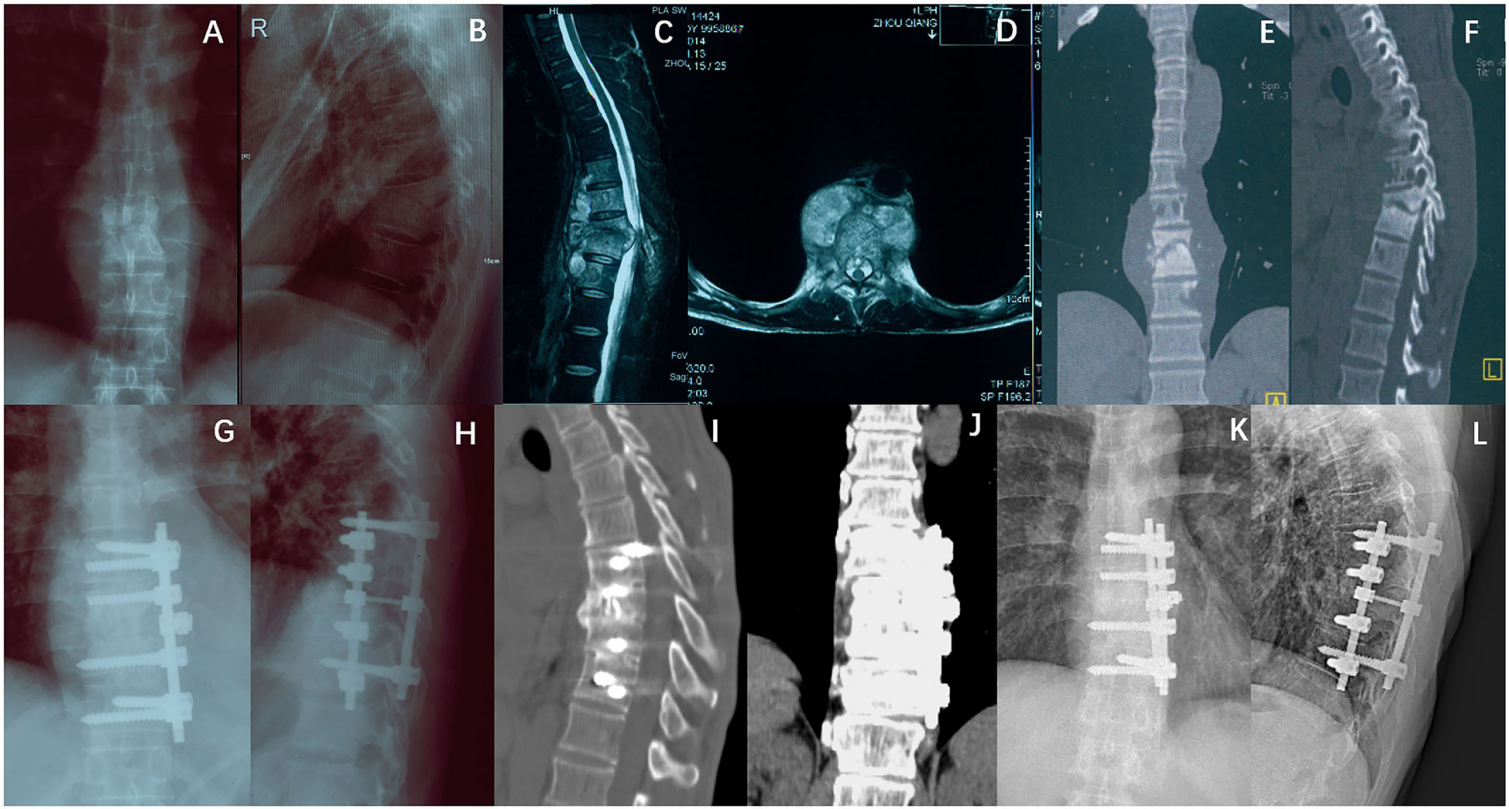
Case II. A 47-year-old male patient with L7–11 TB who was assigned to group B. **a–f** show the preoperative X-ray, CT, and MRI scans. The patient was treated with anterior-only surgery, and the internal fixation region was T8–11. **g** and **h** show the 1-week postoperative X-rays. **i–l** show the 2-year postoperative X-ray, CT, and MRI scans

Typical cases in groups A and B are shown in Figs. 1 and 2, respectively.

## Discussion

Most cases of spinal TB involve 1–2 vertebral bodies; however, many spinal TB cases cannot be diagnosed early in China and other economically underdeveloped regions. Therefore, approximately 9.6% of patients had TB involving more than 1 segment [11]. For patients with multisegment thoracic and lumbar TB, surgery is often required to completely remove the TB lesions, correct the spinal deformity, and stabilize the spine at the same time. The difficulty and risk of surgery are higher for multisegment cases than for single-segment cases. Therefore, selecting the correct surgical method is particularly important.

The main surgical methods of thoracic and lumbar TB include the anterior-only approach, combined anterior and posterior approach, and posterior-only approach [9, 12]. The advantages of the anterior approach include the facts that the lesions can be exposed and removed directly, the deformity can be corrected, and the spine can be stabilized. The results at the long-term follow-up showed that this operation can lead to satisfactory clinical outcomes [13]. However, the anterior approach involves substantial trauma, complex anatomical structures, and a risk of vascular injury [14]. Patients with poor pulmonary function may not be able to tolerate this operation because of the impact on lung function, and the operation for upper thoracic vertebra lesions is difficult because of the obstruction of the sternum and scapula [9, 15]. These deficiencies are more pronounced for multisegment cases that require greater exposure.

Due to multisegment vertebral destruction and collapse, patients with multisegment TB are more likely than those with single-segment TB to develop spinal deformity and instability, and their cases of deformity and instability are often more serious. Some studies have shown that spinal TB cases with more than 2 segments destroyed are associated with a higher risk of kyphosis and failure of bone graft fusion [16, 17]. Moreover, anterior internal fixation is not convenient for cases involving many segments. Therefore, combined anterior and posterior surgery is recommended for severe vertebral destruction or severe kyphosis cases [7]. Mohanty et al. [18] reported that combined anterior and posterior surgery is used to treat T1–L1 TB, as it can effectively correct kyphosis and stabilize the spine while resolving TB. However, combined anterior and posterior surgery has been shown to lead to good clinical results and increased surgical trauma. Memtsoudis et al. [19] reported that the complication rate of combined anterior and posterior surgery was 23.8%, which was significantly higher than that of anterior- or posterior-only surgery, and the mortality rate was nearly twice that of posterior-only surgery. In this study, the complication rate of group B was higher than that of group A, but there was no significant difference.

In recent years, posterior-only surgery has been used for the treatment of thoracic and lumbar TB. Many surgeons believe that posterior-only surgery is suitable for cases of single-segment TB lesions [7, 8] because partial lesion removal cannot be performed under direct vision, which can increase the risk of incomplete lesion clearance. With advancements in the posterior technique, some surgeons have begun to use posterior-only surgery to treat multisegment thoracic TB. Zhong et al. [20] and Wu et al. [21] treated TB patients with multisegment lesions and kyphosis by posterior-only surgery, and all patients were cured and had no recurrence of TB. For multisegment thoracic and lumbar TB, posterior surgery can be performed with multisegment transforaminal and paravertebral approaches according to the type of lesion; in some indirect operations, the lesion surrounding the spine can be effectively cleared, and the case of TB can be cured [22]. Moreover, posterior-only surgery can prevent some complications associated with anterior surgery because the posterior spinal anatomical structure is simple. In this study, all cases were cured without recurrence of TB, so we believe that posterior-only approach surgery, as well as anterior-only approach surgery, is safe and effective in treating consecutive multisegment thoracic and lumbar TB.

The advantages of the posterior approach for the correction of deformities have been recognized by most surgeons [23]. A posterior pedicle screw system can stabilize the three columns of the spine, and pedicle screw placement with the diseased vertebral body can further increase the stability [24, 25]. The posterior approach surgery can provide intervertebral support combined with posterior compression and is more convenient for combined osteotomy, so it has a better ability to correct deformities. Some surgeons consider that anterior-only surgery has been shown to have a limited ability to correct kyphosis, while combined anterior and posterior surgery or posterior-only surgery can lead to good orthopedic results, and posterior-only surgery is superior under proven technical conditions [26]. In this study, we found that the kyphosis correction angle and rate in group A were significantly better than those in group B, and the kyphosis correction loss rate in group B was significantly higher than that in group B. For some patients with severe kyphosis in group A, a satisfactory correction was achieved only by additionally performing 1–2 grade osteotomy. In addition, internal fixation for multisegment TB cases often requires extension of the involved region to adjacent segments, and it is easier to extend the internal fixation region with the posterior approach.

Of course, a variety of surgical methods are available, and surgeons should select surgical strategies according to their skill levels and patients’ pathological characteristics. This study also has some limitations, such as the small number of cases studied and the short follow-up time. The results of this study need to be further confirmed by studies with more cases and long-term follow-ups.

## Conclusion

Posterior-only and anterior-only approach surgery can lead to satisfactory clinical results in the treatment of patients with consecutive multisegment thoracic and lumbar TB. With posterior-only surgery, kyphosis can be better corrected, and the correction can be better maintained than with anterior-only surgery.

## Data Availability

The datasets used and/or analyzed during the current study are available from the corresponding author on reasonable request.

## Abbreviations

ASIA: American Spinal Injury Association
BID: Bis in die
CPR: C-reactive protein
CT: Computed tomography
ESR: Erythrocyte sedimentation rate
IV: Intravenously
MRI: Magnetic resonance imaging
ODI: Oswestry disability index
PO: Per os
QD: Quaque die
TB: Tuberculosis
TID: Ter in die
VAS: Visual analog scale

## References

[1] Wang L, Zhang H, Ruan Y, Chin DP, Xia Y, Cheng S, Chen M, Zhao Y, Jiang S, Du X, He G, Li J, Wang S, Chen W, Xu C, Huang F, Liu X, Wang Y (2014). Tuberculosis prevalence in China, 1990-2010; a longitudinal analysis of national survey data. Lancet..

[2] World Health Organization. Global tuberculosis report. 18 September 2018 Available from: http://www.who.int/tb/publications/global_report/GraphicExecutiveSummary.

[3] Moon MS (1997). Tuberculosis of the spine. Controversies and a new challenge. Spine (Phila Pa 1976).

[4] Huang J, Zhang H, Zeng K, Gao Q (2014). The clinical outcomes of surgical treatment of noncontiguous spinal tuberculosis: a retrospective study in 23 cases. PLoS One.

[5] Cheung WY, Luk KD (2013). Clinical and radiological outcomes after conservative treatment of TB spondylitis: is the 15 years' follow-up in the MRC study long enough?. Eur Spine J.

[6] Issack PS, Boachie-Adjei O (2012). Surgical correction of kyphotic deformity in spinal tuberculosis. Int Orthop.

[7] Yin XH, Liu ZK, Hao D (2018). The reasons and clinical treatments of postoperative relapse of Pott's disease. Medicine (Baltimore).

[8] Zhang HQ, Li JS, Zhao SS, Shao YX, Liu SH, Gao Q, Lin MZ, Liu JY, Wu JH, Chen J (2012). Surgical management for thoracic spinal tuberculosis in the elderly: posterior only versus combined posterior and anterior approaches. Arch Orthop Trauma Surg.

[9] Wang LJ, Zhang HQ, Tang MX, Gao QL, Zhou ZH, Yin XH (2017). Comparison of three surgical approaches for thoracic spinal tuberculosis in adult: minimum 5-year follow up. Spine (Phila Pa 1976).

[10] Zhang P, Peng W, Wang X, Luo C, Xu Z, Zeng H, Liu Z, Zhang Y, Ge L (2016). Minimum 5-year follow-up outcomes for single-stage transpedicular debridement, posterior instrumentation and fusion in the management of thoracic and thoracolumbar spinal tuberculosis in adults. Br J Neurosurg.

[11] Li L, Xu J, Ma Y, Tang D, Chen Y, Luo F, Li D, Hou T, Zhou Q, Dai F, He Q, Zhang Z (2014). Surgical strategy and management outcomes for adjacent multisegmental spinal tuberculosis: a retrospective study of forty-eight patients. Spine (Phila Pa 1976).

[12] Liu P, Sun M, Li S, Wang Z, Ding G (2015). A retrospective controlled study of three different operative approaches for the treatment of thoracic and lumbar spinal tuberculosis: three years of follow-up. Clin Neurol Neurosurg.

[13] Wang B, Lv G, Liu W, Cheng I (2011). Anterior radical debridement and reconstruction using titanium mesh cage for the surgical treatment of thoracic and thoracolumbar spinal tuberculosis: minimium five-year follow-up. Turk Neurosurg.

[14] Hamdan AD, Malek JY, Schermerhorn ML, Aulivola B, Blattman SB, Pomposelli FB (2008). Vascular injury during anterior exposure of the spine. J Vasc Surg.

[15] Zhang H, Sheng B, Tang M, Guo C, Liu S, Huang S, Gao Q, Liu J, Wu J (2013). One-stage surgical treatment for upper thoracic spinal tuberculosis by internal fixation, debridement, and combined interbody and posterior fusion via posterior-only approach. Eur Spine J.

[16] Rajasekaran S, Soundarapandian S (1989). Progression of kyphosis in tuberculosis of the spine treated by anterior arthrodesis. J Bone Joint Surg Am.

[17] Upadhyay SS, Sell P, Saji MJ, Sell B, Yau AC, Leong JC (1993). 17-year prospective study of surgical management of spinal tuberculosis in children. Hong Kong operation compared with debridement surgery for short- and long-term outcome of deformity. Spine (Phila Pa 1976).

[18] Mohanty SP, Pai Kanhangad M, Yogesh Kumar B, Singh A. Single-stage anterior debridement, posterior instrumentation and global fusion in thoracic and thoracolumbar tubercular spondylodiscitis. Musculoskelet Surg. 2018. 10.1007/s12306-018-0581-5 30515742

[19] Memtsoudis SG, Vougioukas VI, Ma Y, Gaber-Baylis LK, Girardi FP (2011). Perioperative morbidity and mortality after anterior, posterior, and anterior/posterior spine fusion surgery. Spine (Phila Pa 1976).

[20] Zhong N, Kong J, Sun Z, Qian M, Liu T, Xiao J (2018). One-stage posterior approach in the treatment of consecutive multi-segment thoracic tuberculosis with kyphosis. Turk Neurosurg.

[21] Wu W, Li Z, Wang S, Zhang H, Lin R, Lin J (2019). One-stage surgical treatment for consecutive multisegment thoracic spinal tuberculosis with kyphosis by posterior-only debridement, interbody fusion, and instrumentation. World Neurosurg.

[22] Zhao C, Pu X, Zhou Q, Huang X, Zhang C, Luo L, Zhang Z, Hou T, Luo F, Dai F, Xu J (2019). Can a posterior approach effectively heal thoracic and lumbar tuberculosis? Microbiology outcomes of the operative area. J Orthop Surg Res.

[23] Yang P, Zang Q, Kang J, Li H, He X (2016). Comparison of clinical efficacy and safety among three surgical approaches for the treatment of spinal tuberculosis: a meta-analysis. Eur Spine J.

[24] Anekstein Y, Brosh T, Mirovsky Y (2007). Intermediate screws in short segment pedicular fixation for thoracic and lumbar fractures: a biomechanical study. J Spinal Disord Tech.

[25] Mahar A, Kim C, Wedemeyer M, Mitsunaga L, Odell T, Johnson B, Garfin S (2007). Short-segment fixation of lumbar burst fractures using pedicle fixation at the level of the fracture. Spine (Phila Pa 1976).

[26] Panchmatia JR, Lenke LG, Molloy S, Cheung KM, Kebaish KM (2015). Review article: Surgical approaches for correction of post-tubercular kyphosis. J Orthop Surg (Hong Kong).

